# Comparison Between Familial Colorectal Cancer Type X and Lynch Syndrome: Molecular, Clinical, and Pathological Characteristics and Pedigrees

**DOI:** 10.3389/fonc.2020.01603

**Published:** 2020-09-02

**Authors:** Yun Xu, Cong Li, Yuqin Zhang, Tian’an Guo, Congcong Zhu, Ye Xu, Fangqi Liu

**Affiliations:** ^1^Department of Colorectal Surgery, Fudan University Shanghai Cancer Center, Shanghai, China; ^2^Department of Oncology, Shanghai Medical College, Fudan University, Shanghai, China

**Keywords:** colorectal cancer, familial colorectal cancer type X, Lynch syndrome, mismatch repair, clinical management

## Abstract

**Objective:**

This study aimed to compare the molecular, clinical, and pathological characteristics and pedigrees of familial colorectal cancer type X (FCCTX) with those of Lynch syndrome (LS) to provide a theoretical basis for the management of FCCTX.

**Methods:**

Overall, 46 cases of FCCTX and 47 LS probands and affected families were enrolled between June 2008 and September 2018 for this study. Multigene cancer panel tests that included 139 genes were performed for all patients, and variants in each group were described. The clinical, pathological, and pedigree characteristics were also compared between the two groups.

**Results:**

In total, 42 variants were detected in 27 (58.7%) cases in the FCCTX group, with *BRCA1*, *BRCA2*, *POLE*, *POLD1*, *ATR*, and *ATM* being the most frequently mutated genes. The mean onset age of colorectal cancer (CRC) was significantly older in the FCCTX group than in the LS group (53.57 ± 12.88 years vs. 44.36 ± 11.26 years, *t* = −9.204, *p* < 0.001). The proportion of patients with rectal cancer was also higher in the FCCTX group than in the LS group [43.5% (20/46) vs. 10.6% (5/47), χ^2^ = 12.823, *p* = 0.005]. Within a median follow-up time of 53.9 ± 37.0 months, the proportion of patients who developed metachronous CRC was significantly higher in the LS group than in the FCCTX group [34.0% (16/47) vs. 13.0% (6/46), χ^2^ = 5.676, *p* = 0.017]. When comparing pedigrees, older age at cancer onset and rectal cancer clustering were observed in the FCCTX families. A higher prevalence in male patients was also observed in the FCCTX families.

**Conclusion:**

FCCTX is an entity distinct from LS, but its genetic etiology remains unknown. A larger multigene panel would be recommended for determining the underlying pathogenic variants. Considering the pathology and moderate penetrance of the CRC link to FCCTX, less stringent surgical treatments and colonoscopy surveillance would be preferable. Rectum preference is a typical feature of FCCTX. Colonoscopy surveillance in FCCTX families could be less intensive, and more attention should be given to male members.

## Introduction

Heredity is a major influencing factor for the occurrence of colorectal cancer (CRC), and approximately 20–30% of CRC patients have a family history of the disease (1). Lynch syndrome (LS) is a dominantly inherited condition characterized by a significantly increased risk for CRC, being the primary etiology in 2–5% of all CRC cases (2, 3). LS arises from pathological variants (PV) in DNA mismatch repair (MMR) genes including *MLH1*, *MSH2*, *MSH6*, and *PMS2* (4). LS can also be caused by MSH2 methylation, which results from defects in *EPCAM* (4).

Clinically, LS is recognized by the Amsterdam criteria (AC), which is formulated according to history-based diagnostic algorithms (5). The introduction of immunohistochemistry (IHC) tests, microsatellite instability analysis, and next-generation sequencing (NGS) within the last decade enabled the development of a more accurate molecular theoretical basis for the identification of LS. Research has shown that a considerable number of families that fulfill the AC neither manifested defects in the MMR protein nor carried PV in MMR genes. As such, it was suggested that this hereditary condition should be considered as a different clinical and genetic entity and was accordingly designated as familial CRC type X (FCCTX) (6).

Familial CRC type X, which is a major subgroup of suspected LS, has a strong background of family history. However, FCCTX phenotypes are different with regard to discrepant molecular etiologies. The management guidelines including for screening, identification, treatment, and surveillance for LS patients and affected families have been well established (7), whereas those for FCCTX cases are yet to be clarified. Accordingly, a complete understanding of the underlying etiology and clinicopathological features would be helpful to develop an appropriate management strategy for FCCTX. Previous analyses of FCCTX patients and associated tumors revealed an older age at onset, moderate penetrance of cancers, and narrow tumor spectrum compared to those in LS patients (8–15). In particular, FCCTX patients have a twofold higher risk of CRC than the general population, which is significantly lower than that of LS patients (6). While other cancers aside from CRC are also frequent in families with LS (16, 17), extracolorectal cancers rarely occur in FCCTX families (6, 8). An analysis of the long-term outcomes of different hereditary CRC subgroups concluded that less frequent but more individualized surveillance protocols would be beneficial for FCCTX cases (18). Nevertheless, these findings were derived from Western medical centers, and data in China remain lacking. Thus, this study aimed to compare the molecular, clinical, and pathological characteristics and pedigrees of FCCTX with those of LS to provide a theoretical basis for the management of FCCTX.

## Materials and Methods

### Study Design and Patients

**FIGURE 1:**
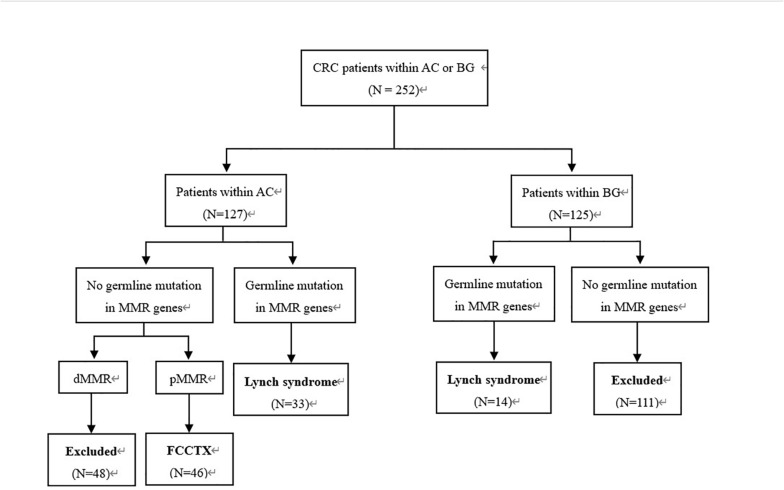
Patient selection flowchart. CRC, colorectal cancer; AC, Amsterdam criteria; BG, Bethesda guidelines; MMR, mismatch repair; FCCTX, familial colorectal cancer type X.

This retrospective study was approved by the institutional review board of our hospital. We evaluated 252 consecutive CRC patients who fulfilled the AC (5) and Bethesda guidelines (19) and underwent curative segmental surgeries according to different tumor loci in our hospital between June 2008 and September 2018. The inclusion criteria were as follows: (a) postoperative pathologically confirmed CRC; (b) detection of PV in MMR genes; (c) pedigrees meeting the AC, but probands carrying no PV of MMR genes; and (d) IHC showing an MMR-proficient (pMMR) profile. A total of 93 patients who met the inclusion were enrolled in this study. The patient selection flowchart is shown in Figure 1.

Immunohistochemistry analyses were performed in all CRC tumors, and multigene cancer panel tests that included 139 genes were performed for all patients. All patients provided informed consent for genetic analyses. Of the 93 patients, 47 identified with PV of the MMR gene were classified as the LS group, and 46 who met the AC and showed a pMMR profile were classified as the FCCTX group.

### Data Collection and Follow-Up

Demographic information, pathologic results, and tumor histories were retrospectively collected. The pedigrees were obtained through patient interviews, and their first and second relatives involving children, siblings, parents, grandparents, aunts, and uncles were recruited. The patient and each relative were asked to report whether that relative had ever been diagnosed with LS-associated cancers, and the clinicodemographic data of those who have diagnosed were recorded. Pathological data of cancers among relatives were systematically collected, if available.

All included patients were followed up every 2 to 3 months. The occurrence of metachronous CRC, distant metastases, and extracolorectal cancer was recorded. Treatment options for these events were determined according to recommendations of our multidisciplinary team. Moreover, incident cases of cancer in their families were recorded, and multigene cancer panel tests were recommended for those patients. These measures could help determine whether they carry the same gene variants and are beneficial for the discovery of novel pathogenic variants. This study was censored on February 25, 2020, and the patients were followed up for a median of 53.9 ± 37.0 months.

### Immunohistochemistry

Mismatch repair gene deletion was determined according to the absence of protein expression for any one of several genes, including hMLH1, hMSH2, hMSH6, and hPMS2. IHC was performed using the fully automated BenchMark ULTRA platform (Ventana Medical Systems, Inc., Tucson, AZ, United States). Normal tissues adjacent to the tumor or lymphocytes in the stroma served as internal positive controls. Each result was confirmed by at least two experienced pathologists.

### Next-Generation Sequencing

Peripheral blood (10 mL) was collected, stored in ethylenediaminetetraacetic acid tubes, and allowed to stand at 25°C for 2 h. The supernatant was transferred to a 15-mL centrifuge tube and then centrifuged for 10 min at 2,200*g* at 4°C. Thereafter, the intermediate white blood cells were transferred to a 1.5-mL centrifuge tube. The DNA was recovered using the MagPure FFPE DNA LQ Kit (Magen). NGS was conducted on the germline DNA as a standard genetic testing for germline analysis.

DNA quantification was performed using the Qubit 2.0 Fluorimeter with the dsDNA HS assay kits (Life Technologies, Carlsbad, CA, United States). A minimum of 50 ng of DNA was required for NGS library construction. DNA shearing was performed using Covaris M220, followed by end repair, phosphorylation, and adaptor ligation. Fragments measuring 200–400 bp were selected using AMPure beads (Agencourt AMPure XP Kit), followed by hybridization with capture probes baits, hybrid selection with magnetic beads, and PCR amplification. The quality and size range of amplified fragments were then assessed by performing bioanalyzer high-sensitivity DNA assay. Paired-end sequencing of the indexed samples was performed on a NextSeq 500 sequencer (Illumina, Inc., United States).

Sequence data were mapped to the reference human genome (hg19) using BWA aligner 0.7.10. Local alignment optimization was performed using GATK 3.2. Germline SNVs were identified using Varscan with default parameters. Germline indels were identified using Varscan and GATK. Pathogenic variants were determined by a clinical molecular geneticist according to the guidelines of the American College of Medical Genetics. ClinVar and Enigma were used during manual curation for final confirmation of the results. The InSIGHT database was used for the pathogenicity classification of the MMR genes. The raw sequencing data have been uploaded to NCBI database (BioProject ID: PRJNA644236).

### Statistical Analysis

Continuous variables were reported as mean ± standard deviation. Differences in categorical variables and continuous variables between the two groups were analyzed using the χ^2^ test or Fisher exact test and Student *t* test, respectively. All statistical analyses were performed using SPSS version 21.0 software (SPSS, Chicago, IL, United States). Two-tailed *p* < 0.05 was considered to indicate statistical significance.

## Results

### Molecular Characteristics

In the LS group, PVs of *MLH1* were identified in 17 (36.2%) probands and those of *MSH2*, *MSH6*, and *PMS2* were identified in 18 (38.3%), 10 (21.3%), and 2 (4.2%) probands, respectively. In patients identified with PVs in MMR genes, the results of IHC MMR staining were consistent with those of gene detection.

In the FCCTX group, 42 variants in 20 genes, namely, *APC*, *ALK*, *ATM*, *ATR*, *AXIN2*, *BRCA1*, *BRCA2*, *BUB1B*, *BLM*, *BRIP1*, *CDH1*, *CHEK2*, *GALNT12*, *MLH3*, *MUTYH*, *NTRK1*, *POLE*, *POLD1*, *PTCH1*, and *RAD50*, were detected in 27 (58.7%, 27/46) individuals, with *BRCA1*, *BRCA2*, *POLE*, *POLD1*, *ATR*, and *ATM* being the most frequently mutated genes. No variant was identified in 19 (41.3%, 19/46) patients. Of the 42 variants, two cases of monoallelic variants in *MUTYH* (*NC_000001.10:g.45797972G*>*A; p.Gln267Ter*) were identified as pathogenic, and one case of frameshift variant in *ATM (NC_000011.10:g.108257512_108257513CT*; *p.Leu762fs*) was identified as a likely pathogenic variant. In addition, 38 variants were identified as variants of uncertain significance (VUS), and one was benign. The detected variants and related demographic characteristics of the 27 patients in the FCCTX group in whom these mutations were detected are summarized in Table 1.

**TABLE 1:** Detected mutations in the 27 patients of the FCCTX group.

**Gender**	**Age**	**Location**	**Gene**	**Variant**	**Variants type**	**Variant impact**
Female	45	Rectum	*ATM*	*NC_000011.10:g.108257512_108257513CT(p. Leu762fs)*	frameshift	Likely pathogenic
Male	58	Rectum	*BRCA1*	*NC_000017.11:g.43094020C*>*T(p. Arg504His)*	SNV	Benign
Male	60	Sigmoid (left)	*POLE*	*NC_000012.11:g.133219820A*>*G(p. Val1514Ala)*	SNV	VUS
Male	72	Descending colon (left)	*RAD50*	*NC_000005.10:g.132588846A*>*G(p. Gln404Arg)*	SNV	VUS
Female	60	Ascending colon (right)	*ATM*	*NC_000011.10:g.108250816C*>*T(p. Arg451Cys)*	SNV	VUS
Male	68	Ascending colon (right)	*BRCA2*	*NC_000013.11:g.32356461T*>*C(p. Ile2490Thr)*	SNV	Benign
Male	75	Ascending colon (right)	*POLD1*	*NC_000019.9:g.50905089T*>*C(p. Val124Ala)*	SNV	VUS
			*POLE*	*NC_000012.11:g.133202349G*>*A(p. Ala2180Val)*	SNV	VUS
			*POLE*	*NC_000012.11:g.133237641C*>*T(p. Ala992Thr)*	SNV	VUS
Female	42	Ascending colon (right)	*ATR*	*NC_000003.11:g.142172064G*>*C(p. Thr2556Ser)*	SNV	VUS
Male	47	Rectum	*ATR*	*NC_000003.11:g.142281919G*>*A(p. Arg109Trp)*	SNV	VUS
			*POLD1*	*NC_000019.10:g.50402703G*>*A(p. Arg311His)*	SNV	VUS
Male	76	Descending colon (left)	*BRIP1*	*NC_000017.10:g.59763416T*>*C(p. Ile896Val)*	SNV	VUS
			*RAD50*	*NC_000005.10:g.132588846A*>*G(p. Gln404Arg)*	SNV	VUS
Female	45	Sigmoid (left)	*BRCA2*	*NC_000013.10:g.32910678T*>*C(p. Ile729Thr)*	SNV	VUS
Female	68	Ascending colon (right), sigmoid (left)	*BARD1*	*NC_000002.11:g.215610562C*>*T(p. Arg565His)*	SNV	VUS
			*BRCA2*	*NC_000013.10:g.32913723G*>*T(p. Ser1744Ile)*	SNV	VUS
Female	43	Ascending colon (right)	*BLM*	*NC_000015.10:g.90769546A*>*G(p. Lys839Glu)*	SNV	VUS
			*BRCA1*	*NC_000017.11:g.43106514G*>*A(p. Leu52Phe)*	SNV	VUS
Male	61	Rectum	*APC*	*NC_000005.9:g.112173705G*>*A(p. Arg805Gln)*	SNV	VUS
Male	73	Rectum	*MUTYH*	*NC_000001.10:g.45797972G*>*A(p. Gln267Ter)*	Stop gained	Pathogenic?
Male	51	Rectum	*MLH3*	*NC_000014.8:g.75515169_75515171del(p. Ile397del)*	Deletion	VUS
Male	53	Sigmoid (left), rectum	*BUB1B*	*NC_000015.9:g.40504755G*>*A(p. Arg814His)*	SNV	VUS
Female	44	Rectum	*MUTYH*	*NC_000001.10:g.45797972G*>*A(p. Gln267Ter)*	Stop gained	Pathogenic
Male	72	Rectum	*GALNT12*	*NC_000003.11:g.37089020C*>*T(p. Pro240Leu)*	SNV	VUS
Male	37	Ascending colon (right)	*NTRK1*	*NC_000005.9:g.112173705G*>*A(p. Arg780Gln)*	SNV	VUS
Male	70	Ascending colon (right)	*PTCH1*	*NC_000009.12:g.95467197T*>*C(p. Ser761Gly)*	SNV	VUS
Female	36	Rectum	*CDH1*	*NC_000016.9:g.68846047A*>*G(p. Thr340Ala)*	SNV	Benign
Male	45	Rectum	*CDH1*	*NC_000016.9:g.68856080C*>*G(p. Leu630Val)*	SNV	Benign
Male	47	Rectum	*ATR*	*NC_000003.11:g.142281919G*>*A(p. Arg109Trp)*	SNV	VUS
Female	43	Ascending colon (right)	*ALK*	*NC_000002.11:g.29448339C*>*T(p. Gly1054Ser)*	SNV	VUS
			*CHEK2*	*NC_000022.10:g.29107934C*>*T(p. Ser252Asn)*	SNV	VUS
Male	48	Ascending colon (right)	*MLH3*	*NC_000014.8:g.75515169_75515171del(p. Ile397del)*	Deletion	VUS
Male	50	Ascending colon (right)	*AXIN2*	*NC_000017.10:g.63537574G*>*A(p. Pro353Leu)*	SNV	VUS

*SNV, single nucleotide variant; VUS, variants of uncertain significance; FCCTX, familial colorectal cancer type X.*

### Clinicodemographic Characteristics

The clinicodemographic characteristics of the 93 enrolled patients are shown by group in Table 2. There were significant differences in the earliest onset age of CRC and primary CRC location between the two groups. The mean onset age of CRC was significantly younger in the LS group than in the FCCTX group (44.36 ± 11.26 vs. 53.57 ± 12.88, *t* = −9.204, *p* < 0.001). Furthermore, the proportion of patients with early onset cancer (i.e., age < 50 years at cancer onset) was significantly higher in the LS group [74.5% (35/47) vs. 50% (23/46), χ^2^ = 5.930, *p* = 0.015]. The proportions of right- and left-sided colon cancer were 38.3% (18/47) and 42.6% (20/47), respectively, in the LS group, and this was higher than that in the FCCTX group at 23.9% (11/46) and 26.1% (12/46), respectively. In contrast, the proportion of rectal cancer patients was significantly higher in the FCCTX group [43.5% (20/46) vs. 10.6% (5/47), χ^2^ = 12.823, *p* = 0.005].

**TABLE 2:** Clinicodemographic characteristics of the 93 colorectal cancer patients by proband.

**Characteristic**	**LS group (*N* = 47)**	**FCCTX group (*N* = 46)**	**χ^2^/*t*-value**	***p*-Value**
Gender			1.428	0.232
Male	26(55.3%)	31(67.4%)		
Female	21(44.7%)	15(32.6%)		
Age (years)^a^	44.36 ± 11.26	53.57 ± 12.88	−9.204	<0.001
<50	35(74.5%)	23(50.0%)	5.930	0.015
≥50	12(2.5.5%)	23(50.0%)		
CEA (ng/ml)			2.461	0.117
<5.2	7(14.9%)	13(28.3%)		
≥5.2	40(85.1%)	33(71.7%)		
CA19-9 (u/ml)				
<40	11(23.4%)	7(15.2%)	0.998	0.318
≥40	36(76.6%)	39(84.8%)		
Primary CRC location			12.823	0.005
Right colon	18(38.3%)	11(23.9%)		
Left colon	20(42.6%)	12(26.1%)		
Rectum	5(10.6%)	20(43.5%)		
Multiple	4(8.5%)	3(6.5%)		
Multiple tumors			1.521	0.217
Occurrence	12(25.5%)	7(15.2%)		
Absence	35(74.5%)	39(84.8%)		
Tumor size^a^ (cm)	5.17 ± 2.61	4.35 ± 2.02	1.703	0.092
Pathological classification			8.943	0.011
Adenocarcinoma	34(72.3%)	42(91.3%)		
Adenocarcinoma with partial mucinous adenocarcinoma	5(10.7%)	4(8.7%)		
Mucinous adenocarcinoma	8(17.0%)	0(0.0%)		
Differentiation			7.839	0.020
Well differentiated	1(2.1%)	2(4.4%)		
Moderately differentiated	28(59.6%)	38(82.6%)		
Poorly differentiated	18(38.3%)	6(13.0%)		
Cancerous node			3.196	0.074
Occurrence	2(4.3%)	7(15.2%)		
Absence	45(95.7%)	39(84.8%)		
Vascular invasion			0.056	0.813
Occurrence	8(17.0%)	7(15.2%)		
Absence	39(83.0%)	39(84.8%)		
Perineural invasion			0.002	0.968
Occurrence	6(12.8%)	6(13.0%)		
Absence	41(87.2%)	40(87.0%)		
T stage			0.932	0.628
T1	7(14.9%)	9(19.6%)		
T2	8(17.0%)	5(10.9%)		
T3	32(68.1%)	32(69.5%)		
N stage				
N0	34(72.3%)	32(69.6%)	1.118	0.572
N1	9(19.1%)	7(15.2%)		
N2	4(8.6%)	7(15.2%)		
Metastasis			0.323	0.570
Occurrence	2(4.3%)	1(2.2%)		
Absence	45(95.7%)	45(97.8%)		
TNM stage			0.492	0.921
I	13(27.7%)	10(21.7%)		
II	17(36.2%)	20(43.5%)		
III	15(31.8%)	15(32.6%)		
IV	2(4.3%)	1(2.2%)		

*^*a*^These data are presented as mean ± standard deviation; other values are presented as number of patients followed by percentage in parentheses. CRC, colorectal cancer; LS, Lynch syndrome; FCCTX, familial colorectal cancer type X.*

### Pathological Characteristics

There were significant differences in pathological classification (χ^2^ = 8.943, *p* = 0.011) and differentiation of CRC tumors (χ^2^ = 7.839, *p* = 0.020) between the two groups. The proportions of partial mucinous and mucinous adenocarcinoma were 10.7% (5/47) and 17.0% (8/47), respectively, in the LS group, and this was significantly higher than those in the FCCTX group at 8.7% (4/46) and 0% (0/46), respectively. The proportion of patients with poorly differentiated tumors was also higher in the LS group [38.3% (18/47) vs. 13.0% (6/46)]. The pathological characteristics of the CRC tumor are summarized by group in Table 2.

### Colorectal Cancer and Progression

Within a median follow-up time of 53.9 ± 37.0, 34.0% (16/47) of patients in the LS group and 13.0% (6/46) of patients in the FCCTX group developed metachronous CRC, with the difference being significant (χ^2^ = 5.676, *p* = 0.017). The interval period between the first and the second CRC was significantly shorter in the LS group than in the FCCTX group (28.78 ± 29.14 months vs. 59.10 ± 28.79 months, *t* = −2.380, *p* = 0.018). The mean incidence of CRC was higher in the LS group (1.55 ± 0.75 vs. 1.22 ± 0.47). Overall, 42.6% (20/47) of patients in the LS group experienced synchronous or metachronous CRC, which is significantly higher than that of 19.6% (9/46) in the FCCTX group (χ^2^ = 4.069, *p* = 0.032).

The distributions of CRC tumors according to primary tumor sidedness were also assessed. The LS group had a significantly higher proportion of patients with right-sided [59.6% (28/47) vs. 34.8% (16/46), χ^2^ = 5.732, *p* = 0.017] and left-sided CRC [63.8% (30/47) vs. 34.8% (16/46), χ^2^ = 7.784, *p* = 0.005] than did the FCCTX group. In contrast, the proportion of rectal cancer patients was significantly higher in the FCCTX group [52.2% (24/46) vs. 17.0% (8/47), χ^2^ = 11.219, *p* = 0.001].

Distant metastases were observed in 9 (19.1%) patients in the LS group and in 14 (30.4%) patients in the FCCTX group, with no significant difference (χ^2^ = 1.591, *p* = 0.207). There was also no significant difference in the distant metastasis interval between the two groups (26.46 ± 15.75 months vs. 24.13 ± 21.46 months, *t* = 0.277, *p* = 0.758). The characteristics of tumor history in probands of the two groups are summarized in Table 3.

**TABLE 3:** Tumor characteristics by proband in the 93 patients with colorectal cancer.

**Characteristic**	**LS group (*N* = 47)**	**FCCTX group (*N* = 46)**	**χ^2^/*t*-value**	***p*-Value**
Earliest onset age of CRC (years)^a^	44.36 ± 11.26	53.57 ± 12.88	−3.671	<0.001
Total number of CRCs^a^	1.55 ± 0.75	1.22 ± 0.47	2.607	0.011
Metachronous CRC	16(34.0%)	6(13.0%)	5.676	0.017
Synchronous or metachronous CRC	20(42.6%)	9(19.6%)	4.609	0.032
Distant metastasis	9(19.1%)	14(30.4%)	1.591	0.207
Right colon cancer	28(59.6%)	16(34.8%)	5.732	0.017
Left colon cancer	30(63.8%)	16(34.8%)	7.784	0.005
Rectal cancer	8(17.0%)	24(52.2%)	11.219	0.001
Earliest onset age of extra-colorectal cancer (years)^a^*	48.45 ± 12.68	53.38 ± 7.87	−0.967	0.347
Synchronous or metachronous extra-colorectal cancer	11(23.4%)	7(15.2%)	1.203	0.252
Earliest onset age of cancer (years)*	43.40 ± 11.17	52.87 ± 12.31	−3.885	<0.001

*^*a*^These data are presented as mean ± standard deviation; other values are presented as number of patients followed by percentage in parentheses. ^∗^These data are limited to patients who developed extra-colorectal cancer. LS, Lynch syndrome; CRC, colorectal cancer; FCCTX, familial colorectal cancer type X.*

### Extracolorectal Cancers

In the LS group, 11 patients (15 cases) developed primary extracolorectal cancers (five cases of endometrial cancer, five cases of gastric cancer, two cases of small intestinal cancer, one case of ovarian cancer, one case of breast cancer, and one case of cutaneous cancer). In the FCCTX group, seven patients (seven cases) developed extracolorectal cancers (two cases of endometrial cancer, two cases of breast cancer, one case of gastric cancer, one case of prostate cancer, and one case of pancreatic cancer). A total of 23.4% (11/47) of patients in the LS group and 15.2% (7/46) of patients in FCCTX group developed synchronous or metachronous extracolorectal cancer, with no significant difference (χ^2^ = 1.203, *p* = 0.252).

### Family Pedigrees

**FIGURE 2:**
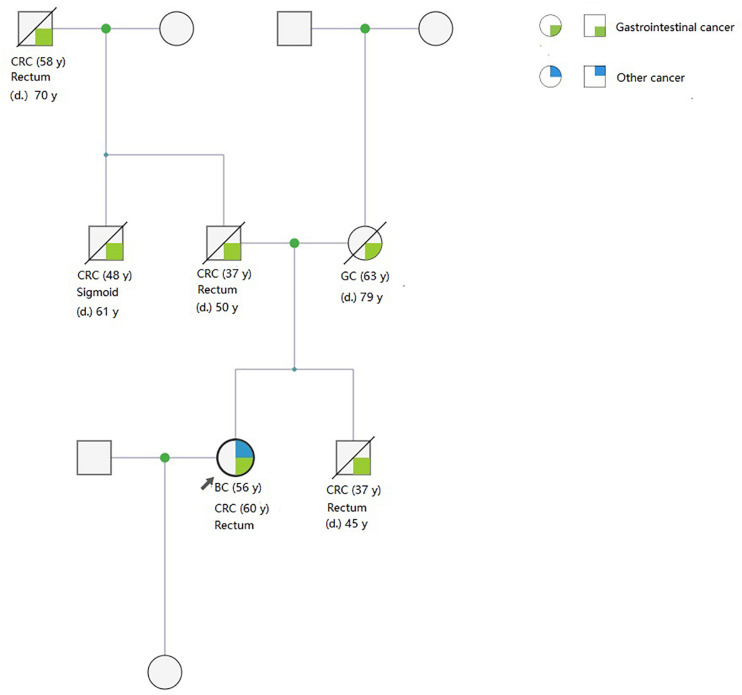
A typical pedigree in the FCCTX group demonstrated rectal cancer clustering. A variant of uncertain significance in *POLD1*: *NC_000019.9:g.50905089T*>*C*(*p. Val124Ala*) was identified in the pedigree. Of the five members who developed CRC, four members had rectal cancer. This may be an overlap phenotype of polymerase proofreading-associated polyposis. CRC, colorectal cancer; GC, gastric cancer; BC, bladder cancer; d., age of death; y, years old; FCCTX, familial colorectal cancer type X.

Pedigrees were determined through interviews and follow-up of enrolled patients and their family members. A total of 142 and 159 first- and second-degree relatives who developed LS-associated cancer in the LS families and in the FCCTX families, respectively, were included in the pedigree analysis. In the comparison of CRC spectrums, significant between-group differences were observed in the numbers of patients who developed left colon cancers and rectal cancer. The mean number of patients who developed left colon cancers was significantly higher in the LS families than in the FCCTX families (1.72 ± 1.19 vs. 1.13 ± 0.86, *t* = 2.746, *p* = 0.007). However, the mean number of patients who developed rectal cancer was significantly higher in the FCCTX families (1.26 ± 0.98 vs. 0.49 ± 0.69, *t* = −4.414, *p* < 0.001). A typical pedigree in the FCCTX group demonstrating rectal cancer clustering is presented in Figure 2. A VUS in *POLD1* was identified in the pedigree. Of the five members who developed CRC, four member had rectal cancer.

The earliest onset age of cancer including those of CRC and extracolorectal cancers was significantly younger in the LS families than in the FCCTX families (36.66 ± 8.75 vs. 40.11 ± 9.26, *t* = −2.147, *p* = 0.036). Furthermore, the rate of synchronous and/or metachronous CRC was also significantly higher in LS families [44.7% (21/47) vs. 21.7% (10/46)]. However, there was no significant difference in the incidence of extracolorectal cancers between the two groups. The pedigrees of the two groups are compared and summarized in Table 4.

**TABLE 4:** Comparison of pedigree between the LS group and the FCCTX group.

**Variable**	**LS group (*N* = 47)**	**FCCTX group (*N* = 46)**	***t*/χ^2^ value**	***p*-Value**
Patients with cancer (cases)^a^	4.02 ± 2.48	4.46 ± 1.79	−0.969	0.335
Male patients(cases)^a^	2.28 ± 1.72	2.72 ± 1.38	−1.365	0.176
Female patients (cases)^a^	1.74 ± 1.42	1.74 ± 1.18	0.020	0.984
First degree relatives (cases)^a^	1.98 ± 1.69	2.04 ± 1.10	−0.219	0.827
Second degree relatives (cases)^a^	1.04 ± 1.55	1.41 ± 1.72	−1.092	0.278
Cases of cancer^a^	5.13 ± 3.10	5.07 ± 1.83	0.118	0.906
Patients with CRC (cases)^a^	3.26 ± 2.08	3.26 ± 1.67	−0.014	0.989
Cases of CRC^a^	3.91 ± 2.54	3.54 ± 1.68	0.829	0.410
Patients with right colon cancer (cases)^a^	1.45 ± 1.02	1.13 ± 1.05	1.479	0.143
Cases of right colon cancer^a^	1.49 ± 1.06	1.13 ± 1.05	1.643	0.104
Right colon cancer			2.200	0.138
Occurrence	38 (80.9%)	31 (67.4%)		
Absence	9 (19.1%)	15 (32.6%)		
Patients with left colon cancer (cases)^a^	1.72 ± 1.19	1.13 ± 0.86	2.746	0.007
Cases of left colon cancer^a^	1.94 ± 1.54	1.15 ± 0.92	2.977	0.004
Left colon cancer			3.181	0.074
Occurrence	43 (91.5%)	36 (78.3%)		
Absence	4 (8.5%)	10 (21.7%)		
Patients with rectal cancer (cases)^a^	0.49 ± 0.69	1.26 ± 0.98	−4.415	<0.001
Cases of rectal cancer^a^	0.49 ± 0.69	1.26 ± 0.98	−4.415	<0.001
Rectal cancer			11.962	0.001
Occurrence	18 (38.3%)	34 (73.9%)		
Absence	29 (61.7%)	12 (26.1%)		
Patients with extra-colorectal cancer (cases)^a^	1.09 ± 1.37	1.46 ± 1.47	−1.263	0.210
Cases of extra-colorectal cancers^a^	1.21 ± 1.49	1.52 ± 1.55	−0.982	0.329
Extra-colorectal cancer			0.666	0.415
Occurrence	30 (63.8%)	33 (71.7%)		
Absence	17 (36.2%)	13 (28.3%)		
Patients with synchronous or metachronous CRC^a^	0.53 ± 0.75	0.28 ± 0.46	2.137	0.046
Synchronous or metachronous CRC			5.506	0.019
Occurrence	21 (44.7%)	10 (21.7%)		
Absence	26 (55.3%)	36 (78.3%)		
Patients with synchronous or metachronous extra-colorectal cancer^a^	0.40 ± 0.68	0.30 ± 0.59	0.755	0.452
Synchronous or metachronous extra-colorectal cancer			0.739	0.390
Occurrence	15 (31.9%)	11 (23.9%)		
Absence	32 (68.1%)	35 (76.1%)		
Earliest onset age of cancer (years)^a^	36.66 ± 8.75	40.11 ± 9.26	−2.147	0.036
Earliest onset age of CRC (years)^a^	37.53 ± 8.63	41.93 ± 7.77	−2.584	0.011
Earliest onset age of extra-colorectal cancer (years)*	45.00 ± 10.27	45.48 ± 19.96	−0.119	0.905

^*a*^These data are presented as mean ± standard deviation; other values are presented as number of patients followed by percentage in parentheses. * These data are limited to families that developed extra-colorectal cancer. LS, Lynch syndrome; CRC, colorectal cancer; FCCTX, familial colorectal cancer type X.

With regard to the sex distribution, we found a significantly higher mean number of male patients in the FCCTX families (2.72 ± 1.38 vs. 1.74 ± 1.18, *t* = 3.656, *p* < 0.001). In contrast, there was no significant difference in the mean number of male and female cancer patients in the LS families (2.28 ± 1.72 vs. 1.74 ± 1.42, *t* = 1.637, *p* = 0.105).

## Discussion

In addition to the pathology, optimal treatment for hereditary CRC should be based on genetic etiology and molecular phenotype (20, 21). Clinically, the recognition of a hereditary CRC as a distinct entity is defined by a clearly recognizable set of clinicopathological features. LS is defined by a characteristic tumor spectrum and pedigree according to AC (5). However, 21–73% of the families meeting the AC for LS diagnosis, i.e., FCCTX, lack evidence of heritable defects in the MMR (6, 13). In our study, 36.2% (46/127) of probands meeting the AC showed pMMR in the IHC test, consistent with the data in previous reports. With regard to the discrepancies between clinical and pathological diagnosis, more accurate tests such as multigene cancer panel tests and whole-genome or whole-exome studies should be recommended to distinguish FCCTX from LS.

Considering the discrepant molecular basis, FCCTX should be classified as a distinct entity. Although the genetic causes of FCCTX are still unknown, several studies identified some CRC predisposing genes including *BRCA2*, *SEMA4A*, *APC*, *BMPR1A*, *NTS*, *CDH18*, *RPS20*, *GREM1*, *BCR*, *KIF24*, *GALNT12*, *ZNF367*, *HABP4*, *GABBR2*, *TP53*, *SMAD4*, and *BMP4* (11–13, 22–24). Some of those genes were detected in the current study, and the most frequently detected variants *MUTYH*, *BRCA1*, *BRCA2*, *POLE*, *POLD1*, *TP53*, *BARD1*, *APC*, and *BRIP1* were associated with other CRC or polyposis syndromes. These results indicate the pleiotropism of some gene variants, manifesting as some genetic overlap with other hereditary cancer syndromes. For patients carrying variants susceptible to other hereditary cancer syndromes, genetic counseling and regular surveillance should be recommended. However, most of the variants in those genes were identified as VUS, and thus, a larger multigene panel for the subset and functional analysis for these VUS should be performed to determine the underlying genetic etiology.

As a specific subset of hereditary CRC syndrome, the distinct molecular etiology is also manifested in the pathological characteristics of tumors. LS-associated CRCs are characterized by poorly differentiated tumors, mucinous differentiation, and an expanding growth pattern (25). In contrast, a higher proportion of moderately and well-differentiated adenocarcinoma tumors were observed in FCCTX-associated CRC (10, 25). These results indicate the moderate pathological features of FCCTX tumors.

Although FCCTX was defined as a distinct entity from LS, the specific management criteria for FCCTX are yet to be established, thus highlighting the importance of the analysis of the clinical features of FCCTX, which can be helpful for developing an effective treatment guideline. In the current study, the onset age was younger in the LS group, whereas the proportion of rectal cancer was significantly higher in the FCCTX group. LS caused by the MMR variant is characterized by markedly increased lifetime risks of cancers at young age (<50 years) (1, 2). Thus, our results implied that FCCTX has moderate cancer penetrance. However, it is noteworthy that 50% of early-onset CRCs were observed in the FCCTX group. A recent study in central Iran reported a high prevalence of FCCTX (77.4%) in early-onset CRCs (26). In some Western medical centers, early-onset age of CRC was considered to be a high-risk factor and an indication for genetic tests (27). In this context, IHC should be routinely performed, and genetic tests should be recommended for the early-onset subgroup. With regard to the CRC tumor location, our study showed a striking clustering in the rectum, consistent with the epidemiology of CRC in China and the findings of previous research (6, 13, 15, 28). These findings indicate that FCCTX-related CRC has a predilection for the rectum. Our previous study has demonstrated that LS in China was characterized by a higher proportion of left colonic cancer (29), and this is supported by the current study findings.

During the follow-up, a significantly higher number of metachronous CRC cases and shorter interval periods were observed in the LS group. Compared with those, metachronous CRC is substantially less likely developed, and this result reconfirmed the moderate penetrance of FCCTX. These findings indicate that the IHC of MMR proteins should be performed in preoperative colonoscopy biopsy, and it might be reasonable to supplement the IHC analysis with NGS or methylation analysis before the decision for colectomy is made. For patients with MMR deficiency, extended colectomy including subtotal colectomy and total colectomy should be recommended, particularly for the patients with synchronous tumors and early-onset age. Furthermore, with regard to surgical indications and techniques for pMMR patients, curative segmental curative resection should remain as the first option, as it can reduce the risk of surgical trauma while ensuring sufficient remnant colon for adequate digestion function and, ultimately, improve the quality of life of FCCTX patients. Moreover, given the lower risk of metachronous CRCs and longer interval between initial and metachronous CRCs in FCCTX, endoscopy can be performed less frequently and in a more individualized schedule. We suggest 5-year surveillance colonoscopies unless findings at the preceding surveillance session indicate the need for a shorter interval (18).

Along with CRC, other cancers such as those in the endometrium, stomach, small bowel, and pancreas have been heavily studied in patients with LS (16, 17). However, the risk of extracolorectal cancers in FCCTX is still controversial, with some studies demonstrating that extracolorectal cancers rarely occur in FCCTX families (6, 8). A Danish report showed significantly increased risks for cancers of the urinary tract, breast, stomach, and pancreas and eye tumors in FCCTX families (30). In our cohort, there were no significant differences in the number of patients who developed extracolorectal cancers between the FCCTX and the LS groups. The differences were in the distribution of these tumors. The most common extracolorectal cancers in LS patients were gastric and endometrial cancers, whereas no specific organ predilection was identified in FCCTX patients. This finding indicates that there may be a genetic overlap with other hereditary cancer syndromes in FCCTX. Thus, regular examinations of other systems could be required during the surveillance.

Family pedigree is the most noticeable characteristic in a distinct hereditary cancer syndrome. Regular screening, surveillances, and genetic counseling for probands and their family members should be conducted for all families with existing diagnosed and suspected hereditary cancer syndromes. Although LS and FCCTX are different entities, they share analogous pedigree features, as found in the current study. The surveillance guidelines for LS are well established (31–33) and are aimed at the diagnosis of colorectal adenomas, early CRC, and other LS-related cancers. Recent clinical guidelines suggest colonoscopy at least once every 2 years, beginning between age 20 and 25 years (32). The Prospective LS Database reported that colonoscopy performed in intervals of <3 years is beneficial for the follow-up of MMR variant carriers (33, 34). Primary prevention screening colonoscopy in asymptomatic family members significantly decreased the risk of CRC in FCCTX (35). Moreover, the screening and surveillance guidelines for FCCTX are yet to be established, thereby limiting recommendations for surveillance. Compared with LS families, the earliest onset age of cancer in FCCTX families was significantly older. Thus, the earliest colonoscopy screening for family members in FCCTX families can be delayed to the earliest onset age in their pedigrees.

Previous studies have reported a higher proportion of rectal cancer in FCCTX (8–11), and consistent findings were found in this study. Although the families presented with a similar phenotype with regard to the distribution of CRC tumors, the harbored variants were diverse, and most tumors were identified as VUS. The pedigree in whom rectal cancer was frequent and met the AC highlights that both LS and FCCTX should be evaluated. Another interesting finding is that there were more males than females in the FCCTX families. The higher prevalence of cancer in male patients in LS families has been previously reported (36), but it is yet to be observed in FCCTX families. The higher prevalence of male cancer patients in the FCCTX families in the current study indicates that more attention should be paid to the screening and surveillance of affected male members.

This study has some limitations. First, the possibility of bias in patient selection could not be eliminated owing to the retrospective nature of the study. Second, not all patients underwent microsatellite instability analysis, thus limiting the robustness of the molecular evidence. Finally, the study had a small sample size, and no variants in PMS2 were found. Further studies with a larger sample size and long-term follow-up are needed. The genetic causes of FCCTX remain unknown, and a larger multigene panel should be conducted to determine the underlying genetic etiology, especially for patients with early-onset CRC. Despite these limitations, we believe that our study remains valuable because, to our best of our knowledge, this is the first study that directly compares LS with FCCTX in Asia, and the results can be helpful to further understand the difference between LS and FCCTX and finally establish the management guidelines for FCCTX.

## Conclusion

In conclusion, FCCTX should be classified as a different entity from LS owing to its distinct molecular features, and specific principles of management should be formulated. FCCTX-associated CRC is characterized by pathological features of a lower proportion of poorly differentiated tumors and mucinous adenocarcinoma. Tumor histories of FCCTX probands showed a moderate penetrance, as evidenced by the older age at cancer onset, less synchronous or metachronous CRC, and a longer interval between the initial and the second CRC. In this context, curative segmental resection and less stringent colonoscopy surveillance should be performed. Given that CRC in both probands and their pedigree had a predilection for the rectum, a pedigree with rectal cancer cluster and meeting AC should be evaluated for both LS and FCCTX. In the pedigree analysis, an older age at cancer onset may indicate that screening endoscopy for affected family members could be delayed. The higher prevalence of male cancer patients in the FCCTX families highlights that more attention should be given to the screening and surveillance of affected male family members.

## Data Availability Statement

The datasets presented in this study can be found in online repositories. The names of the repository/repositories and accession number(s) can be found below: the NCBI BioProject (https://www.ncbi.nlm.nih.gov/bioproject/) (PRJNA644236).

## Ethics Statement

The studies involving human participants were reviewed and approved by the Ethics Committee of the Fudan University Shanghai Cancer Center. The patients/participants provided their written informed consent to participate in this study.

## Author Contributions

YuX, YeX, and FL conceived and designed the study. YuX, CL, YZ, CZ, and TG collected and analyzed the data. All authors contributed to the article and approved the submitted version.

## Conflict of Interest

The authors declare that the research was conducted in the absence of any commercial or financial relationships that could be construed as a potential conflict of interest.
