# Supplementary material for: Comparison Between Familial Colorectal Cancer Type X and Lynch Syndrome: Molecular, Clinical, and Pathological Characteristics and Pedigrees
Source: Front Oncol. 2020 Sep 2;10:1603. doi: 10.3389/fonc.2020.01603 (PMC7493642; doi:10.3389/fonc.2020.01603)
Supplement: Supplementary file 1 [file Table_1.docx]

**Supplementary file 1**. 139 genes in the multigene panel.

AIP, ALK APC, ATM, ATR AXIN2, BAP1, BRAD1, BLM, BMPR1A, BRCA1, BRCA2, BRIP1, BUB1B, CBL, CDC73, CDH1, CDK4, CDKN1B, CDKN1C, CDKN2A, CEBPA, CHEK1, CHEK2, CYLD, DDB2, DICER1, DIS3L2, EGFR, ELANE, EPCAM, ERCC1, ERCC2, ERCC3, ERCC4, ERCC5, EXT1, EXT2, EZH2, FANCA, FANCB, FANCC, FANCD2, FANCE, FANCF, FANCG, FANCI, FANCL, FANCM, FAS, FH, FLCN, GAINT12, GATA2, GEN1, GJB2, GPC3, GREM1, HMBS, HNF1A, HOXB13, HRAS, KIT, LAPS1, MAX, MC1R, MEN1, MET, MITF, MLH1, MLH3, MRE11A, MSH2, MSH6, MTAP, MTUS1, MUTYH, NBN, NF1, NF2, NSD1, NTRK1, PALB2, PALLD, PDGFRA, PHOX2B, PMS1, PMS2, POLD1, POLE, POLH, PPM1D, PRKAR1A, PRSS1, PTCH1, PTCH2, PTEN, PTPN11, RAD50, RAD51B, RAD51C, RAD51D, RB1, RECQL, RECQL4, RET, RHBDF2, RUNX1, SBDS, SDHA, SDHAF2, SDHB, SDHC, SDHD, SLX4, SMAD4, SMARCA4, SMARCB1, SMARCE1, SOS1, STAT3, STK11, SUFU, TERT, TGFBR1, TMEM127, TP53, TSC1, TSC2, UROD, USHBP1, VEGFA, VHL, WRN, WT1, XPA, XPC, XRCC2, ZMAT3.
